# Association between shift work and the risk of death from biliary tract cancer in Japanese men

**DOI:** 10.1186/s12885-015-1722-y

**Published:** 2015-10-21

**Authors:** Yingsong Lin, Takeshi Nishiyama, Michiko Kurosawa, Akiko Tamakoshi, Tatsuhiko Kubo, Yoshihisa Fujino, Shogo Kikuchi, for the JACC Study Group

**Affiliations:** 1 Department of Public Health, Aichi Medical University School of Medicine, 1-1 Yazakokarimata, Nagakute, Aichi 4980-1195 Japan; 2 Department of Epidemiology and Environmental Medicine, Juntendo University School of Medicine, Hongo 2-1-1, Bunkyo-ku, Tokyo, 113-8421 Japan; 3 Department of Public Health, Hokkaido University Graduate School of Medicine, North 15, West 7, Kita-ku, Sapporo, 060-8638 Japan; 4 Department of Preventive Medicine and Community Health, University of Occupational and Environmental Health, Japan, 1-1, Iseigaoka, Yahata-nishi-ku, 807-8555 Kitakyushu, Japan

**Keywords:** Shift work, Cohort study, Risk, Hazard ratio, Biliary tract cancer

## Abstract

**Background:**

There is increasing evidence suggesting that shift work involving night work may increase cancer risk.

**Methods:**

We examined the association between working rotating shifts and the risk of death from biliary tract cancer among Japanese men who participated in the Japan Collaborative Cohort Study. Of the 46,395 men recruited, 22,224 men aged 40–65 at baseline (1988–1990) who reported working full-time or were self-employed were included in the present analysis. The study subjects were followed through December 31, 2009. Information regarding occupation and lifestyle factors was collected using a self-administered questionnaire. Cox proportional hazard models were used to estimate the hazard ratio (HR) and 95 % confidence interval (CI) for the risk of death from biliary tract cancer in relation to shift work.

**Results:**

During a mean 17-year follow-up, we observed 94 biliary tract cancer deaths, including 23 deaths from gallbladder cancer and 71 deaths from extrahepatic bile duct cancer. Overall, shift work was associated with a statistically non-significant increase in the risk of biliary tract cancer, with an HR of 1.50 (95 % CI: 0.81-2.77), among rotating shift workers. When the analysis was limited to extrahepatic bile duct cancer, a significant association appeared, with a multivariable-adjusted HR of 1.93 (95 % CI: 1.00-3.72) for rotating shift workers.

**Conclusion:**

Our data indicate that shift work may be associated with increased risk of death from extrahepatic bile duct cancer in this cohort of Japanese men. The association with gallbladder cancer remains unclear because of the small number of deaths.

## Background

Biliary tract cancer, which includes gallbladder and extrahepatic bile duct cancer, is notoriously difficult to diagnose and treat. The prognosis for patients with advanced-stage biliary tract cancer is dismal, with a 5-year survival rate of <10 % [1]. Japan has a relatively higher incidence of biliary tract cancer compared with other developed countries [2]. According to the latest vital statistics, 6498 Japanese people died of gallbladder cancer and 11,711 died of cancers of other (or unspecified) parts of the biliary tract in 2012 [3]. Of the biliary tract cancers, gallbladder cancer is the most common type, and two pathways—chronic inflammation and anomalous pancreatobiliary junction—have been linked with gallbladder cancer risk [4]. However, the risk factors for extrahepatic bile duct cancers remain largely unknown because of the lack of data. Only one prospective cohort study in Japan has reported that cholelithiasis and obesity are associated with risk of extrahepatic bile duct cancer [5].

The association between occupational exposure and biliary tract cancer has been suggested in previous studies [6–8]. Workers engaging in petroleum refining, paper milling, chemical processing, and shoemaking were found to have an increased risk of gallbladder cancer development [6]. Another occupation-related factor that has wide implications for public health is shift work. Approximately 15-20 % of the working population in industrialized countries is estimated to engage in night-shift work [9], and the effect of such a work schedule on health, including on the formation of cancers, has attracted increasing multidisciplinary research attention [10–13]. Experimental studies have shown that the disruption of circadian rhythms as a result of exposure to light at night can promote carcinogenesis in rodents [13]. In contrast, findings from epidemiologic studies on the association between shift work and cancer risk in humans are mixed and inconclusive [14–21], although a positive association was observed for breast, colorectal, prostate, and lung cancers in some previous studies [14–18]. On the basis of sufficient evidence from animal studies and limited evidence from epidemiologic studies, the working group of International Agency for Research on Cancer (IARC) concluded in 2007 that “shift work that involves circadian disruption is most likely carcinogenic to humans. [22]” However, several limitations of the existing epidemiologic studies have also been highlighted, such as inconsistent definitions across studies and crude exposure measurements [23].

To our knowledge, no studies have reported an association between shift work and biliary tract cancer in humans. Moreover, animal studies have shown that circadian dysregulation disrupts bile acid homeostasis [24], the function of which is linked to obesity, type 2 diabetes, and other chronic diseases such as non-alcoholic steatohepatitis[25]. We therefore hypothesized that shift work is associated with an increased risk of biliary tract cancer. The availability of prospective cohort data on a large number of Japanese individuals provided us with a good opportunity to examine these associations.

## Methods

### Study cohort: The JACC study

A detailed description of the Japan Collaborative Cohort Study (JACC Study) has been published elsewhere [26]. Initiated in 1988, the JACC Study is one of the representative cohort studies in Japan. At baseline (1988–1990), 110,585 people (46,395 men and 64,190 women), aged 40–79 years, were enrolled from 45 areas throughout Japan. Informed consent for participation was obtained from each participant in the majority of study areas. However, it was obtained at the group level in a few areas because the concept of informed consent was not popularized during the 1980s in Japan. In that case, the municipality head gave the consent to participation representing the participants living in that area. The ethics committee at the Aichi Medical University School of Medicine approved the JACC Study, including study design, informed consent procedure, and data collection and analysis.

At baseline, all participants completed a self-administered questionnaire that solicited information on demographic characteristics, family history of cancer, medical history, occupation, and lifestyle factors. Regarding occupation, we asked the participants to choose from pre-coded categories: employed, working a part-time job, self-employed, housewife, no occupation, or other.

We followed the study subjects until December 31, 2009 in 35 areas. Because of logistical problems, we discontinued follow-ups prior to December 31, 2009 in 10 areas. Approximately 6 % of the cohort participants moved out of the study area. During the follow-up period, we checked the vital status using resident registry data obtained from the municipalities. We ascertained information on mortality based on the causes of death recorded on death certificates. Biliary tract cancer was classified according to the 10^th^ revision of the International Classification of Disease (ICD), in which C23 represents malignant neoplasm of the gallbladder and C24 represents malignant neoplasm of other (or unspecified) parts of the biliary tract. C24 comprises malignant neoplasms of the extrahepatic bile duct (C24.0), ampulla of Vater (C24.1), overlapping sites of the biliary tract (C24.8), and unspecified parts of the biliary tract (C24.9). Malignant neoplasms of other (or unspecified) parts of the biliary tract can be generally referred to as malignant neoplasms of the extrahepatic bile duct because the majority of malignant neoplasms of other (or unspecified) parts of the biliary tract are malignant neoplasms of the extrahepatic bile duct.

### Study subjects

Men who were 40–65 years of age and who reported working full-time or were self-employed at baseline were included in the present analysis. Men were excluded if they had missing data on occupation or a history of cancer at baseline, which left 22,224 men eligible for the present analysis. We examined the characteristics such as sex, age, body mass index, and cigarette smoking between those subjects and 46,935 men at baseline. Overall there were no significant differences between the two groups.

### Exposure data

Information on shift work was collected on the basis of the question: “Which form of work schedule have you engaged in for your longest occupation?” Men were asked to indicate the most regular schedule they had undertaken among three work schedules: daytime work, permanent nighttime work, or rotating shift work. The rotating shift work may or may not involve night work.

We also collected information on covariates, including age, height, weight, medical history, family history of cancer, cigarette smoking (current, former, never), alcohol consumption (current, former, never), job type (office work, manual work, or other), physical activity at work (sitting, alternate sitting and standing, or standing with/without moving), workplace (indoor, outdoor, or both), level of perceived stress (low, moderate, high, or, very high), educational level, and marriage status. Body mass index (BMI) was calculated from self-reported height and weight.

### Statistical analysis

Person-years of follow-up were calculated for each cohort participant from baseline to December 31, 2009, or to the date of pancreatic cancer death or any other cause, or to the time of moving out of the study area, whichever occurred first. Subjects who died from causes other than pancreatic cancer or who moved out of the study areas were treated as censored.

Cox proportional hazards models were used to estimate HRs and 95 % CIs for the association between shift work and the risk of death from biliary tract cancer. The results are presented in the forms of both age-adjusted HRs and multivariable-adjusted HRs. Potential confounding factors added into the models included age (continuous), BMI (<20, 20–22.4, 22.5-24.9, ≧25.0), history of cholelithiasis (yes, no), history of diabetes (yes, no), alcohol drinking (never, past, current), cigarette smoking (never, past, current), sleep time (continuous), and perceived stress (low, moderate, high). Individuals with missing covariate data were treated as an additional group. We included time-dependent covariates in the Cox models to test the proportional hazard assumption, and we found that the data met the proportionality assumption. All analyses were performed using SAS 9.1 software (SAS Institute, Cary, NC). P values for the statistical tests were two-tailed and were considered to be statistically significant if they were <0.05.

## Results

During an average follow-up period of 17 years, we observed 94 biliary tract cancer deaths, including 23 gallbladder cancer deaths and 71 extrahepatic bile duct cancer deaths. At baseline, daytime workers, permanent nighttime workers, and rotating shift workers accounted for 84.5 %, 4.9 %, and 10.6 % of the cohort population, respectively. Baseline characteristics according to work schedule are shown Table 1. Compared with daytime workers, rotating shift workers were younger, were more likely to be current smokers, and were more likely to report high stress in daily life. In addition, rotating shift workers had shorter sleep times compared with daytime workers.

**Table 1:** Baseline characteristics of the study subjects according to work schedule in the JACC Study

	Daytime work	Permanent nighttime work	Rotating shift work
	(*N* = 18781)	(*N* = 1083)	(*N* = 2360)
Age (Yr)	52.2 ± 7.4	52.0 ± 7.2	50.4 ± 7.2
Body mass index (kg/m^2^)	22.9 ± 3.7	23.2 ± 2.8	23.1 ± 2.7
History of diabetes (%)	4.9	3.5	4.3
History of cholelithiasis (%)	3.2	2.9	3.3
Current smokers (%)	54.6	54.5	57.5
Current drinkers (%)	77.7	72.4	75.4
High perceived stress in daily life (%)	23.2	23.8	30.4
Job type (%)			
Office work	20.0	6.6	14.1
Manual work	52.0	64.4	48.9
Sleep time (Hr)	7.4 ± 1.0	7.3 ± 1.0	7.1 ± 1.0

Plus minus values are mean ± standard devidation

The percentages do not add up to 100 % due to missing values

Overall, we observed a statistically non-significant increase in the risk of biliary tract cancer death associated with rotating shift work (Table 2). After adjustment for potential confounding factors, the HR was 1.50 (95 % CI: 0.81-2.77) among rotating shift workers compared with daytime workers. However, rotating shift work was significantly associated with the risk of extrahepatic bile duct cancer; the multivariate HR was 1.93 (95 % CI: 1.00-3.72). The association between shift work and the risk of gallbladder cancer is not clear because only one death occurred among the rotating shift workers.

**Table 2:** Association between rotating shift work and the risk of death from biliary tract cancer in the JACC Study

Biliary tract cancer						
	Person-years	Deaths	HR1	95 % CI	HR2	95 % CI
Daytime work	322,340	78	1.00		1.00	
Permanent nighttime work	19,565	4	0.81	0.30-2.20	0.86	0.31-2.36
Rotating shift work	41,042	12	1.43	0.78-2.63	1.50	0.81-2.77
Extrahepatic bile duct cancer	Person-years	Deaths	HR1	95 % CI	HR2	95 % CI
Daytime work	322,340	56	1.00		1.00	
Permanent nighttime work	19,565	4	1.13	0.41-3.11	1.19	0.43-3.31
Rotating shift work	41,042	11	1.83	0.96-3.51	1.93	1.00-3.72

HR: harzard ratio; CI: confidence interval

HR1: adjusted for age

HR2: adjusted for age, body mass index, history of cholelithiasis, history of diabetes, cigarette smoking, alcohol drinking, perceived stress, and sleep time

Data on gallbladder cancer were not shown because only one death occurred from rotating shift workers

To remove the potential effect of underlying diseases at baseline on the risk of death from biliary tract cancer, we conducted an additional analysis that excluded all deaths within the first two years of follow-up. The risk estimate remained unchanged; the HR was 1.52 (95 % CI: 0.82-2.81) for biliary tract cancer and 1.97 (95 % CI: 1.02-3.79) for extrahepatic bile duct cancer.

## Discussion

To our knowledge, our study is the first cohort study to address the association between shift work and the risk of death from biliary tract cancer in the general population. We found that men who reported engaging in rotating shift work had a 1.9-fold increase in the risk of death from extrahepatic bile duct cancer. The major strengths of our study are its prospective design and the long follow-up period. Another strength is the fact that we collected detailed information on lifestyle factors, allowing us to control for potential confounding factors such as cigarette smoking, history of cholelithiasis and diabetes, sleep time, and perceived stress level. Our findings add evidence to the existing literature indicating that shift work may be associated with an increased risk of cancer at multiple sites.

Shift work has been implicated in an increased risk of a variety of health problems, including cardiovascular diseases and cancer [27]. In addition to the biliary tract cancer we observed in this study, prospective cohort studies have shown significant associations between shift work and the risk of breast, prostate, endometrial, and colon cancers [14–17]. In addition, findings from a large case-control study conducted in Canada suggested that men who engaged in shift work had an increased risk of cancer at multiple sites, including the lung, bladder, rectum, and pancreas [28].

Despite mounting evidence of a positive association between shift work and cancer, the underlying mechanisms remain to be determined. Evidence from animal and biomarker studies has strongly suggested that a major pathway underlying the carcinogenicity of shift work lies in the suppressive effect of light-at-night on melatonin levels [29–32]. Melatonin is a not only a primary circadian pacemaker but also possesses well-established growth inhibitory and oncostatic properties [33]. Biomarker studies have clearly shown that night shift workers have substantially lower levels of 6-sulfatoxymelatonin, the primary metabolite of melatonin, during night work and daytime sleep [31, 32]. These findings suggest that the chronic reduction in melatonin among night shift workers is an important carcinogenic mechanism. Given the antitumor effect of melatonin, shift work that impacts the melatonin pathway can be broadly applied to explain the incidence of cancer at different sites, including the positive association with biliary tract cancer observed in this study.

We recognize that this study had several limitations. First, one major limitation is the use of simple, unvalidated questionnaire to collect exposure data on shift work in the baseline survey. Because the baseline exposure data were collected 20 years ago when most findings on the association between shift work and cancer had not been reported, we did not add more questions to evaluate various aspects of shift work, such as duration and chronotype. Notably, the importance of duration in determining the strength of the shift work/cancer association has been increasingly recognized. A 2011 meta-analysis found a significant, positive, dose-response relationship between increasing years of shift work and breast cancer risk [34]. It has been shown that genetic polymorphisms in circadian genes may be partially responsible for diurnal preference [35]. Second, we did not collect data on shift work after the baseline. Men who were engaged in shift work may quit during the follow-up period, and men who were not engaged in shift work at baseline may start shift work. We consider that nondifferential misclassifications is possible, and in this circumstance the exposure-outcome association may have been weakened. Third, the outcome measurement was based on the death certificate only, without pathologic confirmation. Although malignant neoplasm of other (or unspecified) parts of the biliary tract can generally be referred to as malignant neoplasm of the extrahepatic bile duct, the possibility of misclassification exists, and this might have introduced some bias. Fourth, although we adjusted for known or suspected risk factors for biliary tract cancer, it is possible that the risk estimates may have been affected by unknown confounders, such as morning preference and hormone levels. Fifth, the results could be due to chance because of the relatively small number of deaths due to biliary tract cancer (especially gallbladder cancer) among shift workers. Finally, data on women were not included in the analysis because there were too few women who worked full time in this cohort. Given that the incidence of gallbladder cancer is consistently higher in women compared with men, additional studies are needed to address the effect of shift work on cancer risk in women.

## Conclusions

Our prospective data indicate that shift work may be associated with the risk of death from biliary tract cancer—especially extrahepatic bile duct cancer—in this cohort of Japanese men. Our findings will need to be replicated in other studies. The role of shift work in the development of biliary tract cancer should be explored in further studies that include data on various aspects of shift work and biomarkers.

## Abbreviations

HR: Hazard ratio
CI: Confidence interval
IARC: International Agency for Research on Cancer
JACC: The Japan Collaborative Cohort Study
ICD: The International Classification of Disease
